# Identifying conditions for elimination and epidemic potential of methicillin-resistant *Staphylococcus aureus* in nursing homes

**DOI:** 10.1186/s13756-016-0130-7

**Published:** 2016-09-22

**Authors:** Nataliya G. Batina, Christopher J. Crnich, David F. Anderson, Dörte Döpfer

**Affiliations:** 1 Department of Industrial and Systems Engineering, University of Wisconsin-Madison, 3270 Mechanical Engineering Building, 1513 University Avenue, Madison, WI 53706 USA; 2 Department of Medicine, University of Wisconsin-Madison, Madison, WI USA; 3 William S. Middleton Veterans Affairs Hospital, 2500 Overlook Terrace, B5112E, Madison, WI 53705 USA; 4 Department of Mathematics, University of Wisconsin-Madison, 617 E B Van Vleck Hall, 480 Lincoln Dr, Madison, WI 53706 USA; 5 Department of Medical Sciences, School of Veterinary Medicine, University of Wisconsin-Madison, 2027 Veterinary Medicine Building, 2015 Linden Dr, Madison, WI 53706 USA

**Keywords:** USA300 MRSA, Non-USA300 MRSA, Colonization, Nursing homes, Epidemic potential, Conditions for MRSA reduction

## Abstract

**Background:**

Residents of nursing homes are commonly colonized with methicillin-resistant *Staphylococcus aureus* (MRSA) but there is a limited understanding of the dynamics and determinants of spread in this setting. To address this gap, we sought to use mathematical modeling to assess the epidemic potential of MRSA in nursing homes and to determine conditions under which non-USA300 and USA300 MRSA could be eliminated or reduced in the facilities.

**Methods:**

Model parameters were estimated from data generated during a longitudinal study of MRSA in 6 Wisconsin nursing homes. The data included subject colonization status with strain-specific MRSA collected every 3 months for up to 1 year. Deterministic and stochastic co-colonization and single-strain models were developed to describe strain-specific dynamics of MRSA in these facilities. Basic reproduction numbers of strain-independent MRSA, non-USA300 and USA300 MRSA were estimated numerically. The impact of antibiotic use in the past 3 months on the prevalence of strain-specific MRSA and associated basic reproduction numbers were evaluated.

**Results:**

Our models predicted that MRSA would persist in Wisconsin nursing homes, and non-USA300 would remain the dominant circulating strain. MRSA eradication was theoretically achievable by elimination of MRSA-positive admissions over the course of years. Substantial reductions in MRSA prevalence could be attained through marked increase in clearance rates or reduction in MRSA-positive admissions sustained over years. The basic reproduction number of strain-independent MRSA was 0.18 (95 % CI = 0.13–0.23). Recent antibiotic use increased the prevalence of strain-specific MRSA and associated basic reproduction numbers, but was unlikely to lead to an outbreak.

**Conclusions:**

Based on our model, MRSA elimination from nursing homes, while theoretically possible, was unlikely to be achieved in practice. Decolonization therapy that can sustain higher clearance rates or lower MRSA-positive introductions over years may reduce strain-specific prevalence of MRSA in the facilities, and antibiotic stewardship may contribute to this effort. Large-scale MRSA outbreaks were unlikely in this setting.

## Background

Methicillin-resistant *Staphylococcus aureus* (MRSA) is a major cause of healthcare- and community-associated infections [1]. Infections caused by MRSA are more costly to treat and are associated with excess morbidity and mortality compared to infections caused by methicillin-sensitive strains of *S. aureus* [2, 3]. The dynamics of MRSA in hospitals has been explored in a number of studies [4–6] but its dynamics in other settings remains poorly understood. Up to 50 % of nursing home residents are colonized with MRSA [7–9] and these facilities may play an important role in regional spread of this pathogen [10, 11]. While mathematical models have previously been used to describe the role of nursing homes in the regional spread of MRSA [11–14], we are aware of only one study in which intra-facility dynamics of MRSA was modeled in nursing homes [15]. In the latter study, mathematical modeling was used to explain the relative contribution of healthcare workers and residents to the spread of MRSA in nursing homes and the impact of some control strategies. Notably, model inputs employed in the latter study were based on data observed in a number of contexts, including non-nursing home settings.

Historically, specific strains – particularly, those designated by the USA100 CDC pulsotype – have been responsible for most of the MRSA observed in acute- and long-term care settings [7, 16]. However, an increasing number of studies suggest that community-associated strains of MRSA (e.g., USA300 MRSA) are becoming more common in healthcare facilities in the U.S. [16, 17]. This is of particular concern as community-associated strains may demonstrate a greater potential for transmission and virulence [18–20] that, when coupled with high levels of resident frailty, may produce more severe outcomes relative to healthcare-associated strains. Furthermore, a number of factors including recent antibiotic use have been implicated as risk factors for MRSA colonization in long-term care facilities [7, 8, 21]. However, their impact on the strain-specific dynamics of MRSA in nursing homes remains unexplained. Previously, we used Markov chain models to determine the ultimate distribution of residents colonized with non-USA300 and USA300 and to assess the influence of MRSA strain-type and potential risk factors on MRSA acquisition in nursing homes [22]. We found that non-USA300 strains would remain dominant in nursing homes. Among the candidate risk factors considered in our study, antibiotic use within the past 3 months was the only one to significantly increase the acquisition rates of strain-independent and non-USA300 MRSA.

The transmission dynamics of non-USA300 and USA300 MRSA in nursing homes remains largely unexplained. For instance, while nursing homes are known as reservoirs of MRSA [10], the risk of MRSA outbreak and the prospects of MRSA reduction or elimination in this setting have not been well-understood. Furthermore, the effect of resident-specific characteristics, such as previous antibiotic use, and facility-specific characteristics, such as admission and discharge of colonized, on epidemic potential of MRSA have not been established. In this work, we focused on studying the epidemic potential of MRSA and the relative merits of interventions to reduce or eliminate MRSA from nursing home facilities using compartmental and stochastic modeling approaches. It should be noted that we focused on the U.S. nursing homes most of which provide a mixture of post-acute, rehabilitative and domiciliary services to residents that are generally elderly and require ongoing skilled nursing care. The specific aims of this study were: (1) to evaluate the impact of selected measures on the prevalence of strain-specific MRSA over time and to determine the conditions for MRSA elimination from study nursing homes; (2) to assess the epidemic potential of MRSA and its sensitivity to changes in acquisition and clearance rates, and admission and discharge of colonized; (3) to assess the impact of antibiotic use in the previous 3 months on the prevalence of strain-specific MRSA and its outbreak potential.

## Methods

### Overview

Deterministic compartmental models were developed to describe the transmission dynamics of MRSA in the study nursing homes. Stochastic models were subsequently simulated to assess the impact of randomness on predicted outcomes. The study was reviewed and approved by the Health Sciences Institutional Review Board of the University of Wisconsin-Madison.

### Data

All model inputs, including model parameters, were derived from data collected during a longitudinal study of antibiotic resistance in six nursing homes in Wisconsin [23]. MRSA surveillance cultures were collected from multiple anatomical locations of each subject at baseline and every 3 months thereafter for a period of up to 1 year [23]. The subject’s MRSA status was considered non-evaluable if a culture from any of the routinely screened body sites was missing. Thus, 446 out of 449 subjects contributed evaluable observations to this study. It should be noted that the baseline assessment was not evaluable for two subjects with multiple observations over the study period [22]. Their assessment at 3 months was considered as baseline assessment for the purpose of this study. Data on antibiotic use in the previous 3 months, dichotomized into *No* or *Yes*, were abstracted from health records at the time surveillance cultures were performed. Most of the antibiotics were administered orally and were prescribed for treating urinary tract infection. About 15 % of antibiotics, including sulfonamides and tetracyclines, may have possessed anti-MRSA activity. The data from the six study nursing homes were merged to improve the precision of results; that is, the study outcomes are representative of the hypothetical “combined” Wisconsin nursing home. Facility-level differences in characteristics of these nursing homes and MRSA colonization trends in individual facilities were described elsewhere [22, 23].

### Model overview

**Fig. 1:**
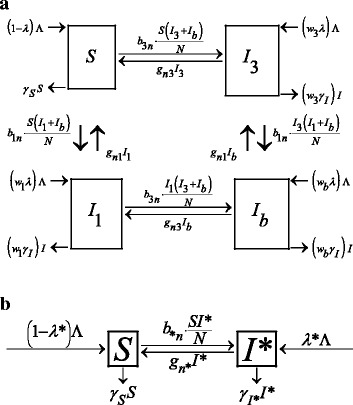
Model diagrams. Compartmental models which describe transmission dynamics of MRSA in nursing homes. The rectangles represent cohorts of residents; the arrows represent inflow to and outflow from each compartment due to disease transmission, clearance, admission or discharge, including death. **a** Co-colonization model (model parameters are described in Table 1), *S* is the cohort of residents susceptible to any MRSA; *I*_*1*_ is the cohort of residents colonized with non-USA300 and susceptible to USA300; *I*_*3*_ is the cohort of residents colonized with USA300 and susceptible to non-USA300; *I*_*b*_ is the cohort of residents colonized with both MRSA strains; **b** Model for colonization with a single MRSA strain-type, either non-USA300 or USA300 MRSA, where λ*, γ_I*_, b_*n_, *g*_n*_, and I* represent the corresponding parameters specific to each single strain model, non-USA300 or USA300

Strain-specific transmission was studied in a co-colonization model (i.e., a model that describes colonization with non-USA300, USA300 and simultaneous colonization with both strains of MRSA) which was considered in both deterministic and stochastic frameworks (Fig. 1a). Deterministic models provide a simple mechanism for describing the dynamics of MRSA in the population on average and for assessing the outbreak potential of the pathogen. However, predictions from deterministic models may lack accuracy when applied to populations with few subjects in either compartment, like ours, and do not account for variability in the predicted outcomes. Subsequently, to enhance our understanding of the transmission mechanism, a stochastic co-colonization model was implemented.

The co-colonization model in a deterministic framework was used to assess the relative merits of selected interventions, the outbreak potential of MRSA, and the impact of antibiotic use in the past 3 months on MRSA prevalence and a possibility of outbreak. To evaluate the relative contribution of each strain-type to the outbreak potential of MRSA, single-strain models in a deterministic framework were also considered (Fig. 1b).

In all models, the number of contacts between residents was assumed to be independent of the size of the nursing home (this assumption regarding the underlying structure of contacts is referred to as frequency-dependent transmission or mass action [24]). Colonization was assumed to occur via random mixing between colonized and non-colonized residents. No distinction was made between colonized and infected. The entire dataset was used to implement every model. The computation was performed in R, version 3.1.0 and higher [25].

### Transmission dynamics of MRSA and impact of selected measures on its prevalence over time

In the co-colonization model, the study population was divided into four mutually exclusive compartments: susceptible to any MRSA (*S*), colonized with non-USA300 only (*I*_*1*_), colonized with USA300 only (*I*_*3*_) and co-colonized with both strains (*I*_*b*_) (Fig. 1a). In the deterministic model, the total population size, *N*, was assumed to stay constant over time to reflect the fixed nursing home capacity and 100 % bed occupancy: *N = S(t) + I*_*1*_*(t) + I*_*3*_*(t) + I*_*b*_*(t)*. In the stochastic model, the population size was assumed not to exceed *N*. The constant *N* was estimated as the number of subjects with at least one evaluable observation over the study period. The model parameters and initial conditions are described in detail in Table 1. The probability of an admitted resident to be colonized, λ, was estimated as the proportion of colonized residents at baseline. The discharge rate of colonized and non-colonized residents, *γ*_*I*_ and γ_S_, were estimated as inversely proportional to the length of stay for colonized and non-colonized, respectively. The length of stay was calculated as an average period of time between residents’ admission dates and their baseline examinations, taken across the six facilities. The discharge rates were assumed to include the discharge from the facility and death from any cause. The admission rate (Λ) was assumed to equal the discharge rate at any time *t*: Λ(*t*) = *γ*_*S*_*S*(*t*) + *γ*_*I*_*I*(*t*), where *I*(*t*) = *I*_1_(*t*) + *I*_3_(*t*) + *I*_*b*_(*t*). The acquisition and clearance rates for non-USA300 (*b*_*1n*_ and *g*_*n1*_) and USA300 (*b*_*3n*_ and *g*_*n3*_) were estimated as transition rates between non-colonized and colonized with the corresponding strain when fitting 2 two-state continuous-time Markov chain models [26] described elsewhere [22]. The states of these models represented non-colonized and colonized with a single MRSA strain irrespective of colonization with the other strain. The rates represent relative frequency per 3 months. The R package *msm* (version 1.3 or above) was used to fit each model to the entire dataset [25, 27]. Acquisition and clearance of one strain was assumed to be independent from carriage of the other strain. The following system of non-linear differential equations described the deterministic co-colonization model:

**Table 1:** Parameters and initial occupancy for the co-colonization model

Description	Symbol	Value (95 % CI)	Estimated or Observed
Initial occupancy in each compartment			
Number of residents susceptible to any MRSA	*S*	350	observed
Number of residents colonized with non-USA300	*I*_*1*_	79	observed
Number of residents colonized with USA300	*I*_*3*_	14	observed
Number of residents co-colonized with non-USA300 and USA300	*I*_*b*_	3	observed
Total number of residents	*N*	446	observed
Model Parameters			
Probability of admission of MRSA-colonized residents	_*λ*_	0.215	estimated
Discharge rate of susceptibles (per 3 months)	*γ*_*S*_	0.101	estimated
Discharge rate of MRSA-colonized (per 3 months)	*γ*_I_	0.074	estimated
Proportion of residents colonized with non-USA300 at baseline	*w*_1_	0.823	observed
Proportion of residents colonized with USA300 at baseline	*w*_3_	0.146	observed
Proportion of residents co-colonized with both strains at baseline	*w*_b_	0.031	observed
Acquisition rate for non-USA300^a^	*b*_*1n*_		
- general^b^		0.029 (0.022, 0.038)	estimated
- w/o AB^c^		0.020 (0.014, 0.030)	estimated
- with AB^d^		0.042 (0.030, 0.061)	estimated
Acquisition rate for USA300^a^	*b*_*3n*_		
- general^b^		0.006 (0.004, 0.011)	estimated
- w/o AB^c^		0.004 (0.002, 0.010)	estimated
- with AB^d^		0.009 (0.005, 0.019)	estimated
Clearance rate for non-USA300^a^	*g*_*n1*_		
- general^b^		0.116 (0.088, 0.152)	estimated
- w/o AB^c^		0.125 (0.086, 0.179)	estimated
- with AB^d^		0.107 (0.074, 0.163)	estimated
Clearance rate for USA300^a^	*g*_*n3*_		
- general^b^		0.137 (0.082, 0.228)	estimated
- w/o AB^c^		0.155 (0.079, 0.328)	estimated
- with AB^d^		0.125 (0.064, 0.266)	estimated

*AB*, antibiotic use in the past 3 months

^a^Relative frequency per 3 months

^b^The rate irrespective of antibiotic use in the past 3 months

^c^The rate corresponding to *No* level of antibiotic use in the past 3 months

^d^The rate corresponding to *Yes* level of antibiotic use in the past 3 months

1 $$ \left\{\begin{array}{c}\hfill \frac{dS}{dt}=\left(1-\lambda \right)\varLambda -{b}_{1n}\left({I}_1+{I}_b\right)\cdot \frac{S}{N}-{b}_{3n}\left({I}_3+{I}_b\right)\cdot \frac{S}{N}+{g}_{n1}{I}_1+{g}_{n3}{I}_3-{\gamma}_SS,\hfill \\ {}\hfill \frac{d{I}_1}{dt}={w}_1\lambda \varLambda +{b}_{1n}\left({I}_1+{I}_b\right)\cdot \frac{S}{N}-{b}_{3n}\left({I}_3+{I}_b\right)\cdot \frac{I_1}{N}-{g}_{n1}{I}_1+{g}_{n3}{I}_b-{w}_1{\gamma}_II,\ \hfill \\ {}\hfill \frac{d{I}_3}{dt}={w}_3\lambda \varLambda +{b}_{3n}\left({I}_3+{I}_b\right)\cdot \frac{S}{N}-{b}_{1n}\left({I}_1+{I}_b\right)\cdot \frac{I_3}{N}-{g}_{n3}{I}_3+{g}_{n1}{I}_b-{w}_3{\gamma}_II,\ \hfill \\ {}\hfill \frac{d{I}_b}{dt}={w}_b\lambda \varLambda +{b}_{1n}\left({I}_1+{I}_b\right)\cdot \frac{I_3}{N}+{b}_{3n}\left({I}_3+{I}_b\right)\cdot \frac{I_1}{N}-{g}_{n1}{I}_b-{g}_{n3}{I}_b-{w}_b{\gamma}_II.\ \hfill \end{array}\right. $$

The stability of endemic and disease-free equilibria [24] (the state of the system when no changes are observed over time, while the disease is present or absent, respectively) was established by dynamically running the system of differential Eqs. (1) with the initial conditions (Table 1) to steady-state using the *rootSolve* package in R environment [25]. The steady-state tolerance was set to the nearest hundredth.

The sensitivity analysis of the MRSA prevalence to the estimated parameters of the model was performed. The changes in the point prevalence of non-USA300 and USA300 MRSA over time were predicted for the values of admission prevalence, (λ) between 0 and 0.5 with step 0.1. This range includes and exceeds the admission prevalence of MRSA reported in other studies [9, 15, 16]. The ranges for the sensitivity analysis of discharge rates of colonized and non-colonized (γ_I_ and γ_S_) were chosen based on the respective interquartile ranges of resident length of stay across the facilities. Thus, discharge rates of 0.05-1.05 (step 0.2) for colonized and 0.05-0.55 (step 0.1) for non-colonized were employed in the sensitivity analysis. Acquisition rates (*b*_*1n*_ and *b*_*3n*_) up to three times as high as the estimate for non-USA300 and 10 times higher than the estimated rate for USA300 were considered for the sensitivity analysis (that is, 0–0.12 (step 0.02) for non-USA300 and 0–0.06 (step 0.01) for USA300). Clearance rates (*g*_*n1*_ and *g*_*n3*_) between the values twice lower and twice higher than estimated rates were employed in the sensitivity analysis (that is, 0.058–0.232 (step 0.029) for non-USA300 and 0.068–0.278 (step 0.042) for USA300).

The parameters of the deterministic co-colonization model (1) were altered to assess their impact on the strain-specific point prevalence of MRSA in the following scenarios:

- *Scenario 1*: elimination of intra-facility MRSA cross-transmission (*b*_*1n*_ = *b*_*3n*_ = 0; Table 1),
- *Scenario 2*: doubling of the strain-specific MRSA clearance rates (2×[*g*_*n1*_] and 2×[*g*_*n3*_]; Table 1),
- *Scenario 3*: elimination of all MRSA-positive admissions (λ = 0; Table 1),
- *Scenario 4*: elimination of a half of MRSA-positive admissions (λ/2; Table 1).

Elimination of intra-facility cross-transmission (Scenario 1) could theoretically be achieved through active surveillance and adherence to more aggressive forms of contact precautions than the ones commonly employed in nursing homes or decolonization therapy [28, 29]. It should be noted that by cross-transmission we understood transition from being non-colonized to colonized with non-USA300 or USA300 strain of MRSA, or transition from colonized with non-USA300 or USA300 to co-colonized with both strains. Decolonization therapy was shown to increase MRSA clearance rates (Scenario 2) in nursing homes [28]. Elimination or reduction in MRSA-positive admissions (Scenarios 3 and 4) could be achieved through active surveillance at admission coupled with decolonization therapy [28]. This intervention was implemented by some hospitals, and positive results were reported [30, 31]. Furthermore, reducing MRSA prevalence and transmission in the community and healthcare settings that transfer patients to nursing homes, including acute-care hospitals, may reduce the admission prevalence of MRSA in nursing home facilities. The prevalence of MRSA under each scenario may help to inform infection control in nursing homes.

The stochastic co-colonization model was a continuous-time Markov chain model and was simulated using Gillspie’s algorithm [32, 33]. The possible state transitions of the model and their respective rates are presented in Appendix Table A1. The model was simulated 1000 times, and the mean of the 1000 simulations was calculated.

#### Assessing epidemic potential of MRSA

The basic reproduction number, R_0_, was used as a threshold to assess the epidemic potential of MRSA in the study nursing homes. In the context of our study, R_0_ represented the expected number of secondary cases of colonization with strain-independent MRSA caused by a single MRSA-colonized resident in a totally susceptible population [34]. A value of R_0_ greater than 1 would predict the spread of MRSA in the population over time and imply that MRSA may become epidemic. The basic reproduction number was estimated by the Next Generation Method [34], using pertinent parameters from Table 1. Standard errors and 95 % confidence intervals (CI) around point estimates were computed by means of bootstrapping with 1000 resampling runs and assuming approximate normality of standard errors [35].

To evaluate the relative contribution of non-USA300 and USA300 strains to the epidemic potential of MRSA, their basic reproduction numbers (R_01_ and R_03_, respectively) were estimated from two single-strain models (Fig. 1b). In the single-strain models, the population was divided into non-colonized (*S*) and colonized with the strain of interest (I*), where both *S* and I* may have contained subjects colonized with the other strain (i.e., when calculating R_01_, a resident colonized with USA300 but free of non-USA300 would be considered non-colonized). Each single-strain model was described by the system of differential equations:

2 $$ \left\{\begin{array}{l}\frac{dS}{dt}=\left(1-{\lambda}^{*}\right)\varLambda -{b}_{*n}{I}^{*}\cdot \frac{S}{N}+{g}_{n*}{I}^{*}-{\gamma}_SS,\hfill \\ {}\frac{d{I}^{*}}{dt}={\lambda}^{*}\varLambda +{b}_{*n}{I}^{*}\cdot \frac{S}{N}-{g}_{n*}{I}^{*}-{\gamma}_{I^{*}}{I}^{*}\hfill \end{array}\right. $$

where λ*, γ_I*_, b_*n_, *g*_n*_, and I* represent the respective parameters specific to each single strain model, non-USA300 or USA300. For non-USA300 model, the probability of admitting colonized (λ^*^) was estimated to be 0.184, and the discharge rate of colonized (γ_I*_) was 0.081. For USA300 model, these parameters were 0.038 and 0.046, respectively. These strain-specific parameters were calculated using the same procedure as the respective strain-independent parameters from Table 1. The Next Generation Method [34] was used to obtain R_01_ and R_03_, and 95 % confidence intervals were calculated by bootstrapping with 1000 resampling runs [35].

For the co-colonization model (Fig. 1a), the basic reproduction number was examined over the space of acquisition and clearance rates of non-USA300 and USA300 within their 95 % CI’s (Table 1). For that purpose, the 95 % CI’s for the four rates (Table 1) were partitioned into steps of size 0.001, and an R_0_ was recalculated for each possible combination of the rates. Furthermore, the sensitivity of R_0_ to the acquisition and clearance rates, discharge rate of colonized, and probability of MRSA-positive admissions was assessed in a one-way sensitivity analysis in which each parameter was varied between 0 and 1 while keeping all other model parameters at their fixed values.

### Impact of antibiotic use in the previous 3 months

The co-colonization model (Fig. 1a) with antibiotic-specific acquisition and clearance rates (Table 1) was used to estimate the changes in the point prevalence of strain-specific MRSA over time for each level of recent antibiotic use (*Yes* and *No*). The hypothetical “best case” and “worst case” scenarios were examined by using the combinations of upper and lower bounds of the 95 % CI’s around antibiotic-specific acquisition and clearance rates (Table 1). The “worst case” scenarios assumed high acquisition and low clearance, while the “best case” scenarios were low acquisition and high clearance. For each level of antibiotic use, the basic reproduction numbers R_0_, R_01_ and R_03_ were estimated from the co-colonization model (Fig. 1a) and respective single-strain models (Fig. 1b) in which antibiotic-specific acquisition and clearance rates were used (Table 1).

## Results

**Fig. 2:**
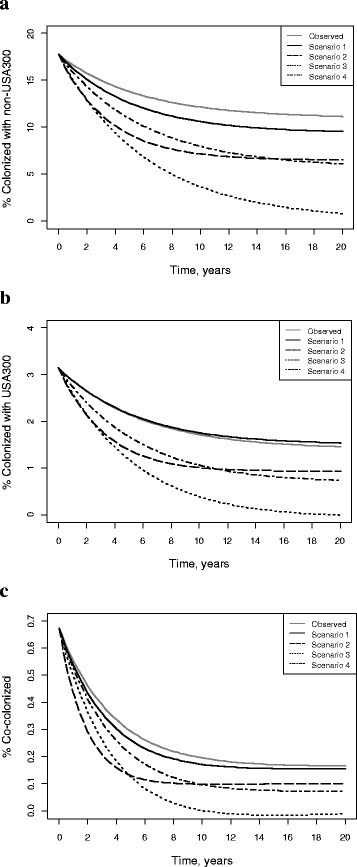
Distribution of subjects colonized with strain-specific MRSA over time under selected scenarios derived from the deterministic model. The curve *Observed* represents the percent of colonized subjects over time based on the observed data; *Scenario 1* shows the percent of colonized subjects under the assumption of no intra-facility cross-transmission; *Scenario 2* reflects the percent of colonized subjects when MRSA clearance rates are increased two-fold; *Scenario 3* represents the percent of colonized over time when all positive MRSA admissions are eliminated; *Scenario 4* shows the percent of colonized over time when a half of all positive MRSA admissions are eliminated. **a** Percent colonized with non-USA300; **b** Percent colonized with USA300; **c** Percent co-colonized with non-USA300 and USA300

Our deterministic model predicted that under the observed conditions, MRSA would remain endemic in our “combined” nursing home over the long run, and non-USA300 would remain the dominant circulating strain (solid line in Fig. 2). The equilibria were stable. After 20 years, 11 %, 1.5 %, and less than 1 % of residents were predicted to be colonized with non-USA300, USA300 and co-colonized with both MRSA strains, respectively. The outcomes of the stochastic simulations highlighted the variability in the MRSA dynamics over time, but trends, in aggregate, were qualitatively similar to the ones of the deterministic model (Appendix Figures A1, A2, and A3).

Elimination of intra-facility cross-transmission had a modest impact on the predicted prevalence of non-USA300 and very little impact on the predicted prevalence of USA300 and co-colonized with both strains (Scenario 1 in Fig. 2). Greater reductions in the prevalence of MRSA could be achieved through doubling clearance rates or reducing the probability of admission of colonized two-fold (Scenarios 2 and 4 in Fig. 2). The only scenarios under which MRSA extinction could be achieved were through elimination of all MRSA-positive admissions (Scenario 3 in Fig. 2) or through selective discharge of colonized residents (Appendix Figure A4). It should be noted, however, that predicted reductions or elimination of MRSA would take years even if any of these unlikely conditions could be achieved.

**Fig. 3:**
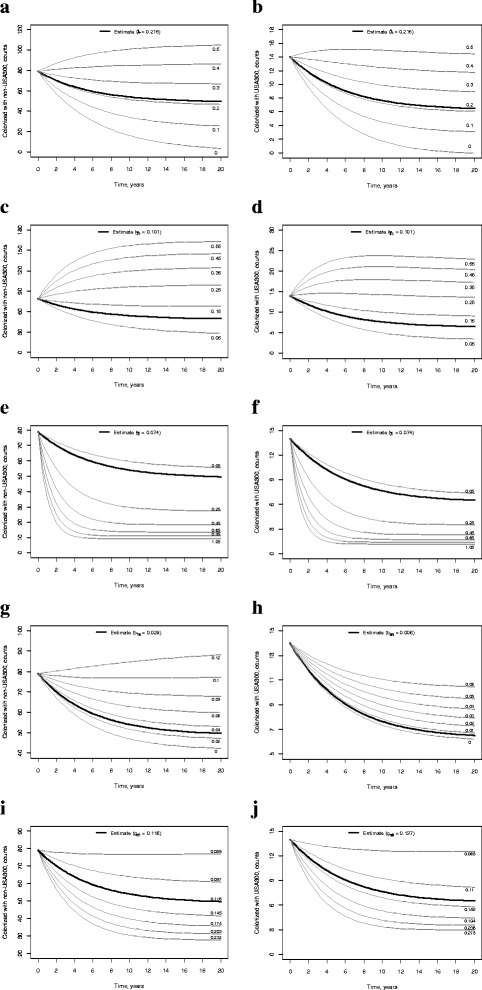
Sensitivity of the point prevalence of MRSA to the estimated parameters. The solid black lines represent the strain-specific point prevalence of MRSA given the estimated model parameter (Table 1). The grey lines represent the point prevalence of MRSA calculated for various values of the parameter; these values are displayed as labels above or below the lines. **a** Sensitivity of the prevalence of non-USA300 to MRSA admission prevalence, λ; **b** Sensitivity of the prevalence of USA300 to MRSA admission prevalence, λ; **c** Sensitivity of the prevalence of non-USA300 to discharge rates of non-colonized, γ_S_; **d** Sensitivity of the prevalence of USA300 to discharge rates of non-colonized, γ_S_; **e** Sensitivity of the prevalence of non-USA300 to discharge rates of colonized, γ_I_; **f** Sensitivity of the prevalence of USA300 to discharge rates of colonized, γ_I_; **g** Sensitivity of the prevalence of non-USA300 to acquisition rates of non-USA300, *b*_*1n*_; **h** Sensitivity of the prevalence of USA300 to acquisition rates of USA300, *b*_*3n*_; **i** Sensitivity of the prevalence of non-USA300 to clearance rates of non-USA300, *g*_*n1*_; **j** Sensitivity of the prevalence of USA300 to clearance rates of USA300, *g*_*n3*_

The point prevalence of non-USA300 and USA300 was sensitive to the admission prevalence of MRSA, λ (Fig. 3a-b). However, the point prevalence of either strain decreased over time for any realistic values of the admission prevalence (admission prevalence as high as 31 % was reported in one nursing home in California [9] but generally lower values were observed [9, 16]). Growth of the strain-specific point prevalence of MRSA over time was predicted for higher values of discharge rates of non-colonized, γ_S_, considered in the sensitivity analysis (Fig. 3c-d). The point prevalence of both non-USA300 and USA300 decreased for the range of discharge rates of colonized, γ_I_, employed in the sensitivity analysis (Fig. 3e-f). Three-fold increase of non-USA300 and 10-fold increase of USA300 acquisition rates (*b*_*1n*_ and *b*_*3n*_, respectively) were predicted to yield decreased point prevalence of the respective strain over time (Fig. 3g-h). Likewise, the point prevalence of either strain was not expected to grow over time if the respective clearance rate (*g*_*n1*_ and *g*_*n3*_) was twice lower than the estimate (Fig. 3i-j). The prevalence of either strain was even less sensitive to the changes in the rates of the other strain.

**Fig. 4:**
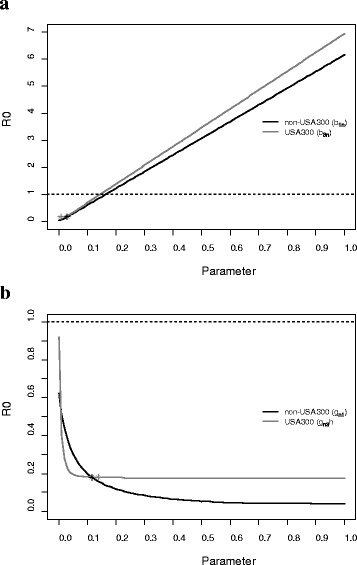
Sensitivity of the basic reproduction number, R_0_, to changes in acquisition and clearance rates, one at a time. The horizontal dotted lines highlight the R_0_ threshold value of 1. The stars display the actual value of R_0_, that is, the value calculated using model parameters (Table 1). **a** Sensitivity of R_0_ to acquisition rates of non-USA300 (*b*_*1n*_) and USA300 (*b*_*3n*_); **b** Sensitivity of R_0_ to clearance rates of non-USA300 (*g*_*n1*_) and USA300 (*g*_*n3*_)

The basic reproduction number for strain-independent MRSA was estimated to be substantially below the threshold of 1 (R_0_ = 0.18; 95 % CI = 0.13 - 0.23) (Table 2). The value of R_0_ did not exceed 0.25 for any combination of acquisition and clearance rates within their 95 % CI’s. The changes in R_0_ within the 95 % CI’s for the pairs of acquisition and clearance rates are illustrated in Appendix Figure A5. The basic reproduction number for non-USA300 (R_01_ = 0.16; 95 % CI = 0.11 - 0.21) was substantially higher than the one for USA300 (R_03_ = 0.04; 95 % CI = 0.01 - 0.06) (Table 2). The sensitivity analysis (Fig. 4) suggested that R_0_ was most sensitive to acquisition rates.

**Table 2:** Basic reproduction number for strain-independent and strain-specific MRSA

Basic Reproduction Number	Point Estimate (95 % CI)		
	General (irrespective of AB)	w/o AB	with AB
Strain-independent MRSA, R_0_	0.18 (0.13, 0.23)	0.12 (0.07, 0.17)	0.28 (0.16, 0.40)
Non-USA300, R_01_	0.16 (0.11, 0.21)	0.11 (0.06, 0.15)	0.24 (0.15, 0.34)
USA300, R_03_	0.04 (0.01, 0.06)	0.02 (0.00, 0.05)	0.05 (0.02, 0.09)

*AB*, antibiotic use in the past 3 months

**Fig. 5:**
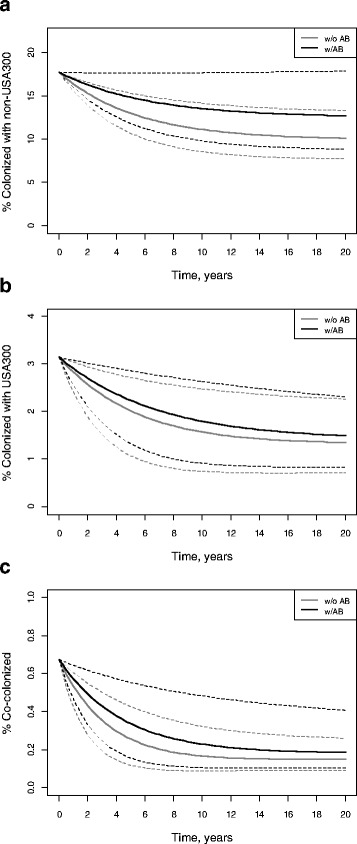
Impact of antibiotic use in the past 3 months on MRSA point prevalence and epidemic potential. Black lines represent the point prevalence of colonized who used antibiotics in the past 3 months, and grey lines show the point prevalence of those who did not. Solid lines denote the distribution of colonized when MRSA acquisition and clearance rates were set at the respective point estimates (Table 1). Dashed lines represent the distribution of colonized in hypothetical scenarios which employed the combinations of upper and lower bounds of the 95 % CI’s of the respective acquisition and clearance rates. Thus, the dashed lines above the corresponding solid lines represent scenarios with high acquisition rates and low clearance rates, while dashed lines below the corresponding solid lines show scenarios with low acquisition and high clearance rates. **a** Distribution of colonized with non-USA300; **b** Distribution of colonized with USA300; **c** Distribution of co-colonized with non-USA300 and USA300

Antibiotic use in the past 3 months was associated with non-significant increases in the point prevalence of non-USA300 and USA300 in the nursing home (Fig. 5). The predicted prevalence of non-USA300 among antibiotic-exposed residents was consistently higher than among unexposed residents over 20 years (Fig. 5a). This difference was less pronounced for USA300 and co-colonized residents (Figs. 5b, c). The estimated value of R_0_ (Table 2) was higher among those who used antibiotics in the past 3 months (R_0_ = 0.28; 95 % CI = 0.16 - 0.40) compared to those who did not (R_0_ = 0.12; 95 % CI = 0.07 - 0.17) although this difference was not statistically significant at a 95 % confidence level. The value of R_01_ (Table 2) was significantly higher for those with the recent history of antibiotic use (R_01_ = 0.24; 95 % CI = 0.15 - 0.34) than for the ones without it (R_01_ = 0.11; 95 % CI = 0.06 - 0.15).

## Discussion

Residents of nursing homes are commonly colonized with MRSA and there is increasing recognition that these facilities influence patterns of MRSA within other healthcare facilities in the same region [36, 37]. Mathematical modeling has been extensively used to understand the dynamics of MRSA within hospitals and its spread between healthcare facilities within the same region [4, 6, 12, 13]. In contrast, attempts to employ mathematical modeling to gain a better understanding of the dynamics of MRSA within the nursing home context remain sparse. The current study advances our knowledge about the prospects of reducing or eliminating MRSA from nursing homes and, conversely, the potential for these facilities to experience a MRSA outbreak. It also examines the impact of recent antibiotic use on the prevalence of MRSA and its outbreak potential.

Our study suggests that elimination of MRSA from nursing homes in the U.S. is highly unlikely under any reasonable conditions. Based on our model, eradication of MRSA could be eventually achieved in nursing facilities if all MRSA-positive admissions were eliminated over a prolonged period of time (years). This intuitive finding is similar to the finding of the other modeling study [15], which concluded that MRSA would persist in nursing homes for as long as colonized residents continue to enter the facilities. Elimination of MRSA introductions could theoretically be achieved through active surveillance, particularly when coupled with effective social distancing interventions or decolonization therapy. Nevertheless, the likelihood of achieving these outcomes in actual practice seems remote for a variety of reasons.

Surprisingly, elimination of intra-facility cross-transmission had a minimal impact on the predicted prevalence of strain-specific MRSA over years. While acquisition rates depend on the intrinsic characteristics of the pathogen, they may also be influenced by colonization pressure [38] and mixing patterns among residents and between residents and staff within the facilities. Several studies have demonstrated that active surveillance, particularly when combined with social distancing measures, can be effective in reducing MRSA prevalence in hospitals and nursing homes [29, 39]. However, the findings of our study suggest that this approach would be of limited utility in nursing homes. This is particularly germane in the long-term care setting where social interactions between residents are considered a goal of care.

Two-fold increase in strain-specific clearance rates or two-fold decrease in MRSA-positive admissions, which could be achieved through the use of decolonization therapy [40], was associated with more substantial reductions in strain-specific prevalence of MRSA if sustained for several years. However, limitations in currently available de-colonization interventions may be a significant barrier to this reduction approach. Intra-nasal mupirocin has been shown to reduce (but not to eliminate) MRSA carriage in several nursing home studies [28, 41]. Coupled with imperfect surveillance for MRSA [7, 42, 43], these reductions may be more moderate than expected. Moreover, these studies demonstrated that de-colonization in most residents was transient and associated with the emergence of resistance [41]. Consequently, the effectiveness of using mupirocin to reduce the prevalence of MRSA in nursing homes over the long run may be limited.

Taken together, our findings suggest that MRSA elimination in nursing homes is unlikely but reductions in the prevalence of MRSA are theoretically achievable, particularly through interventions that accelerate the rate of resident de-colonization or reduce the admission prevalence of MRSA. Nevertheless, our models predict that these interventions would need to be sustained over a period of years in order to have a demonstrable effect on observed patterns of colonization.

An MRSA outbreak in the study nursing homes as quantified by an R_0_ value greater than 1, while theoretically possible, was only achievable under extraordinary circumstances (e.g., multi-fold increase in MRSA acquisition rates). However, minor strain-specific outbreaks were observed during a number of realizations of the stochastic model. Collectively, these findings suggest that while major MRSA outbreaks are unlikely to take place in nursing homes, minor outbreaks may occasionally occur. The basic reproduction number of non-USA300 appeared to be significantly higher than that of USA300 suggesting a higher epidemic potential of non-USA300 strain of MRSA. Furthermore, while recent antibiotic exposure elevated epidemic potential of MRSA, it was unlikely to lead to an outbreak. More research is needed to study factors that may lead to MRSA outbreaks in nursing homes and to evaluate possible outbreak prevention strategies.

The limitations of our study are primarily influenced by the scarcity of data, particularly for USA300 MRSA, and modeling assumptions. For example, while individual nursing homes may differ in transmission dynamics of MRSA, we combined the data from the six facilities due to its paucity in individual facilities. Hence, the results would be applicable to a hypothetical “combined” nursing home. On the other hand, in our previous work we found that the patterns of predicted prevalence at steady state in most facilities mirrored the patterns for pooled data [22].

The standard assumption of random mixing among residents may not be realistic in nursing homes where residents tend to socialize selectively, may share a room, may have limited mobility or be bed-bound. Even though our homogeneous-mixing model predicted that MRSA outbreaks were unlikely in the study nursing homes, they may actually be occurring in subgroups of residents (e.g., in subgroups with substantially higher contact rates between colonized and susceptibles). Our simplifying assumption of constant acquisition and clearance rates of strain-specific MRSA may not be true in nursing homes. The reasons include the variability in contact patterns among residents and between residents and staff, differences in exposure to potential risk factors, colonization pressure [38] and the effect of super-spreaders [44]. Furthermore, we assumed that transmission and acquisition of either strain was independent of colonization status with the other strain, while there is no evidence in favor or against this assumption. Moreover, our models did not account for MRSA transmission through hands of health care workers which may play a role in the spread of MRSA in nursing homes [15, 45]. Additionally, the admission prevalence of MRSA that was assumed to be constant in our models may vary over time. The reasons include differences in the prevalence of MRSA colonization among residents admitted from hospitals and the community.

A number of model parameters were not readily available in our dataset and were estimated from our data. Thus, the point prevalence of MRSA at baseline was used as admission prevalence, though these values may differ in practice. One study of ten nursing homes in California found that the point prevalence of MRSA, while being significantly higher, correlated well with the admission prevalence in the study facilities [9]. MRSA admission prevalence reported in this study varied between 8 % and 31 %, with half of the facilities admitting 21 % or more of MRSA colonized [9]. Our estimate of 0.215 is comparable with MRSA admission prevalence reported in that study, but is higher than those in other studies [15, 16]. It is also likely to be an overestimate for our study facilities. If so, our results may be a worse prognosis than reality. On the other hand, if the length of stay for non-colonized is lower than our estimate, then our prognosis may be too optimistic. Based on our sensitivity analysis, realistic changes in other estimated parameters are unlikely to substantially alter the predicted prevalence. Given a limited knowledge of MRSA patterns in nursing homes, our study is an important step in continuum research that aims at improving our understanding of the dynamics of strain-specific MRSA in nursing homes.

To our knowledge, our study is the first modeling study that sought to assess the epidemic potential of strain-specific MRSA, to evaluate the conditions for MRSA elimination, and to examine the relative merits of selected reduction scenarios of stain-specific MRSA in nursing homes. More research is needed to assess other potential conditions for reducing MRSA colonization in nursing homes and evaluate their cost-effectiveness. Modeling techniques that accommodate heterogeneity of mixing patterns among residents may be a valuable tool in this effort. Furthermore, examining the associations between the candidate risk factors for MRSA colonization and MRSA pulsotypes differentiated at a higher strain similarity threshold may aid in better informing the choice of interventions aimed at reducing the burden of MRSA in nursing homes in the U.S.

## Conclusions

We used mathematical modeling to assess the outbreak potential of MRSA colonization in nursing homes and to evaluate conditions for eliminating or reducing MRSA in this setting. Our study suggests that despite the presence of USA300 strains in a number of the study nursing homes, non-USA300 will remain the dominant circulating MRSA strain in Wisconsin facilities. We found that MRSA elimination from the U.S. nursing homes, while theoretically possible, is unlikely to be achieved in practice. Marked reductions in the strain-specific MRSA in this setting may be attained by using active surveillance coupled with decolonization therapy that can sustain substantially higher clearance rates or reductions in MRSA admission prevalence over years, and antibiotic stewardship may contribute to this effort. Eliminating intra-facility cross-transmission had little impact on the predicted prevalence of MRSA in our models. Despite the recent emergence of novel MRSA strains, including USA300, our models predicted that large-scale outbreaks were unlikely in the nursing home setting.

## Figures and Tables

**Fig. 6:**
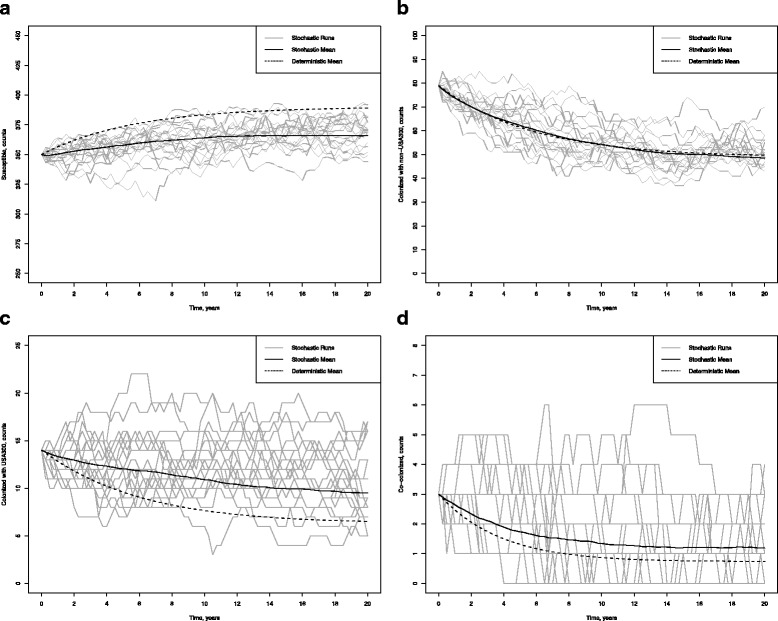
Counts of strain-specific MRSA over time in the hypothetical “combined” nursing home based on stochastic and deterministic models. Solid black lines represent means of the 1000 realizations of the stochastic model; grey lines show the first 20 individual runs of the stochastic model. Dashed black lines are counts derived from the deterministic model. **a** counts of subjects susceptible to any MRSA strain; **b** counts of subjects colonized with non-USA300 and susceptible to USA300; **c** counts of subjects colonized with USA300 and susceptible to non-USA300; **d** counts of subjects co-colonized with non-USA300 and USA300

**Fig. 7:**
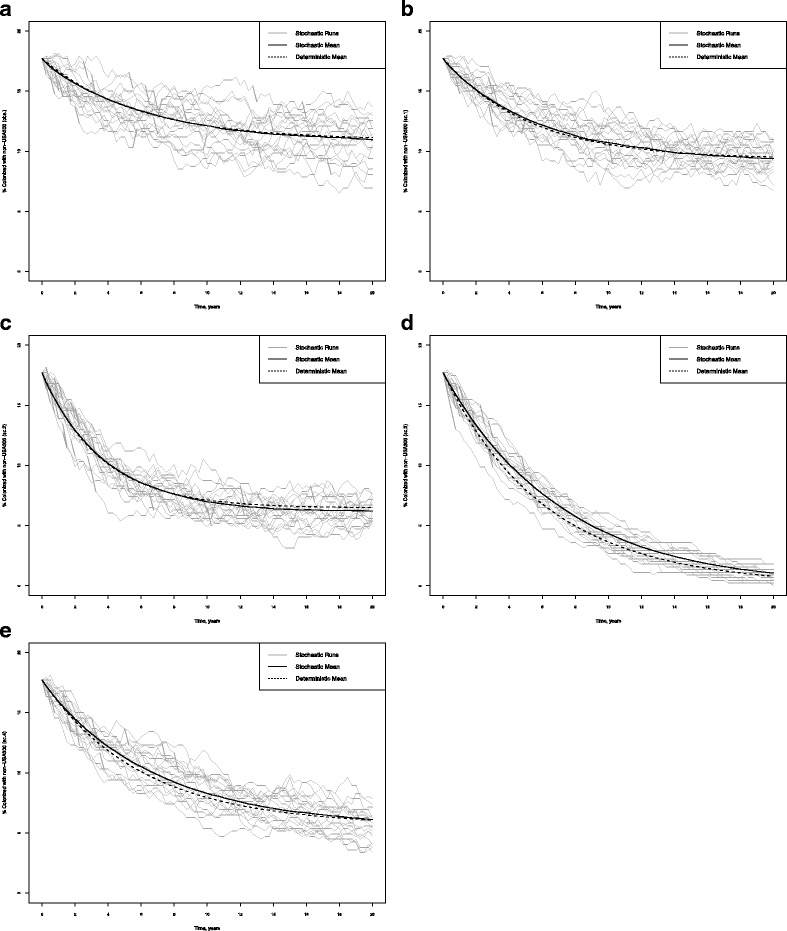
Distribution of subjects colonized with non-USA300 over time in selected scenarios. The black dotted lines represent the percent colonized with non-USA300 derived from the deterministic model. The black solid line represents the mean of 1000 stochastic simulations, and the grey solid lines show the first 20 out of 1000 individual simulations. **a** Predictions based on observed data; **b** Predictions under the assumption of no intra-facility cross-transmission of MRSA; **c** Predictions under the assumption of two-fold increase of clearance rates of MRSA; **d** Predictions when the influx of colonized into the facility is prevented; **e** Predictions when the influx of colonized into the facilities is reduced two-fold

**Fig. 8:**
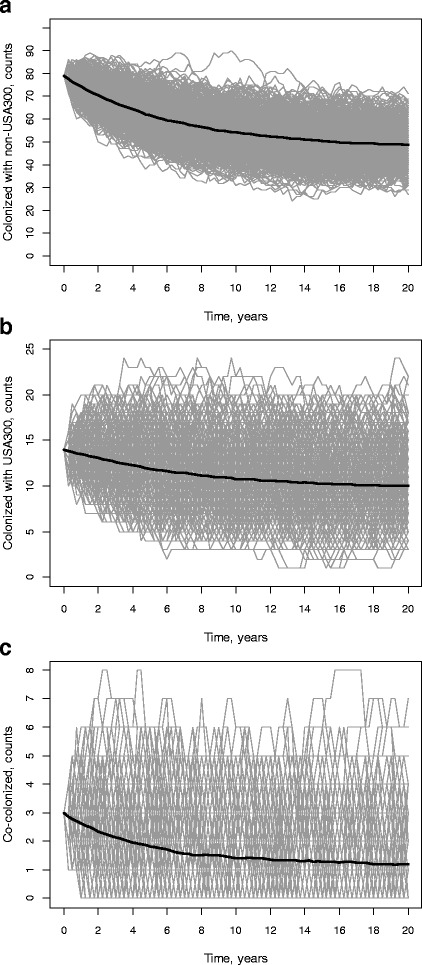
Counts of strain-specific MRSA over time in the hypothetical “combined” nursing home based on the stochastic model. Solid black line represents the mean of the 1000 realizations of the stochastic model; grey lines show all 1000 individual runs of the stochastic model. **a** counts of subjects colonized with non-USA300 and susceptible to USA300; **b** counts of subjects colonized with USA300 and susceptible to non-USA300; **c** counts of subjects co-colonized with non-USA300 and USA300

**Fig. 9:**
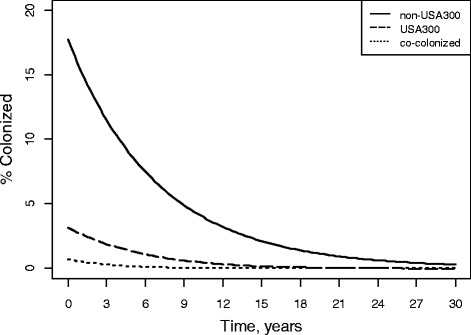
Distribution of subjects colonized with strain-specific MRSA over time under the hypothetical assumption of selective discharge of colonized residents. The curves *non-USA300*, *USA300 and co-colonized* represent the percent of subjects colonized with non-USA300, USA300 and co-colonized with both strains, respectively

**Fig. 10:**
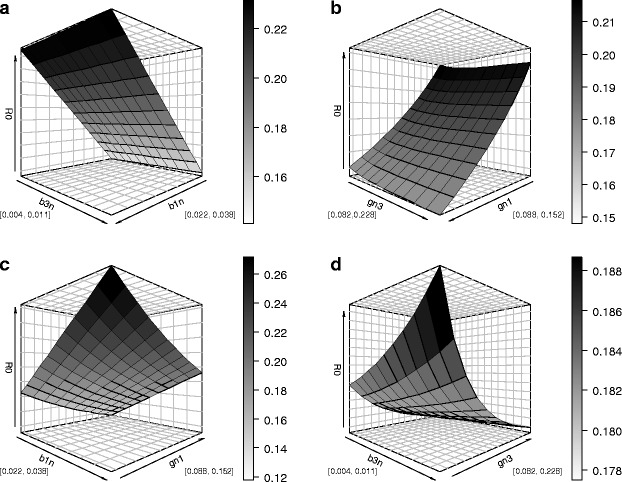
Basic reproduction number of strain-independent MRSA, R_0_, within 95 % confidence intervals of estimated acquisition and clearance rates of strain-specific MRSA. **a** R_0_ within 95 % CI’s of acquisition rates of non-USA300 (*b*_*1n*_) and USA300 (*b*_*3n*_); **b** R_0_ within 95 % CI’s of clearance rates of non-USA300 (*g*_*n1*_) and USA300 (*g*_*n3*_); **c** R_0_ within 95 % CI’s of acquisition and clearance rates of non-USA300 (*b*_*1n*_ and *g*_*n1*_, respectively); **d** R_0_ within 95 % CI’s of acquisition and clearance rates of USA300 (*b*_*3n*_ and *g*_*n3*_, respectively)

## Appendix

**Table 3:** Transitions between the states of the stochastic model

Event	Transition	Rate Constant	Rate
Admission of susceptible residents	∅ → *S*	(1 − *λ*)Λ	(1 − *λ*)Λ
Discharge of susceptible residents	*S* → ∅	*γ*_*S*_	*γ*_*S*_ *S*
Admission of colonized with non-USA300	∅ → *I*_1_	(*w*_1_ *λ*)Λ	(*w*_1_ *λ*)Λ
Discharge of colonized with non-USA300	*I*_1_ → ∅	*w*_1_ *γ*_*I*_	(*w*_1_ *γ*_*I*_)*I*
Transmission of non-USA300 from colonized with non-USA300 to susceptible	*S* + *I*_1_ → 2*I*_1_	$\frac{b_{1n}}{N}$	$\frac{b_{1n}}{N}S{I}_1$
Transmission of non-USA300 from co-colonized to susceptible	*S* + *I*_*b*_ → *I*_1_ + *I*_*b*_	$\frac{b_{1n}}{N}$	$\frac{b_{1n}}{N}S{I}_b$
Clearance of colonized with non-USA300	*I*_1_ → *S*	*g*_*n*1_	*g*_*n*1_ *I*_1_
Admission of colonized with USA300	∅ → *I*_3_	(*w*_3_ *λ*)Λ	(*w*_3_ *λ*)Λ
Discharge of colonized with USA300	*I*_3_ → ∅	*w*_3_ *γ*_*I*_	(*w*_3_ *γ*_*I*_)*I*
Transmission of USA300 from colonized with USA300 to susceptible	*S* + *I*_3_ → 2*I*_3_	$\frac{b_{3n}}{N}$	$\frac{b_{3n}}{N}S{I}_3$
Transmission of USA300 from co-colonized to susceptible	*S* + *I*_*b*_ → *I*_3_ + *I*_*b*_	$\frac{b_{3n}}{N}$	$\frac{b_{3n}}{N}S{I}_b$
Clearance of colonized with USA300	*I*_3_ → *S*	*g*_*n*3_	*g*_*n*3_ *I*_3_
Admission of co-colonized with both strains	∅ → *I*_*b*_	(*w*_*b*_ *λ*)Λ	(*w*_*b*_ *λ*)Λ
Discharge of co-colonized with both strains	*I*_*b*_ → ∅	*w*_*b*_ *γ*_*I*_	(*w*_*b*_ *γ*_*I*_)*I*
Transmission of USA300 from co-colonized to colonized with non-USA300	*I*_1_ + *I*_*b*_ → 2*I*_*b*_	$\frac{b_{3n}}{N}$	$\frac{b_{3n}}{N}{I}_1{I}_b$
Transmission of USA300 from colonized with USA300 to colonized with non-USA300	*I*_1_ + *I*_3_ → *I*_*b*_ + *I*_3_	$\frac{b_{3n}}{N}$	$\frac{b_{3n}}{N}{I}_1{I}_3$
Transmission of non-USA300 from co-colonized to colonized with USA300	*I*_3_ + *I*_*b*_ → 2*I*_*b*_	$\frac{b_{1n}}{N}$	$\frac{b_{1n}}{N}{I}_3{I}_b$
Transmission of non-USA300 from colonized with non-USA300 to colonized with USA300	*I*_1_ + *I*_3_ → *I*_1_ + *I*_*b*_	$\frac{b_{1n}}{N}$	$\frac{b_{1n}}{N}{I}_3{I}_1$
Clearance of co-colonized from USA300	*I*_*b*_ → *I*_1_	*g*_*n*3_	*g*_*n*3_ *I*_*b*_
Clearance of co-colonized from non-USA300	*I*_*b*_ → *I*_3_	*g*_*n*1_	*g*_*n*1_ *I*_*b*_

## Abbreviations

CI: confidence interval
MRSA: Methicillin-resistant *Staphylococcus aureus*

## References

[1] Dantes R, Mu Y, Belflower R, Aragon D, Dumyati G, Harrison LH (2013). National burden of invasive methicillin-resistant Staphylococcus aureus infections, United States, 2011. JAMA Intern Med.

[2] Capitano B, Leshem OA, Nightingale CH, Nicolau DP (2003). Cost effect of managing methicillin-resistant Staphylococcus aureus in a long-term care facility. J Am Geriatr Soc.

[3] Anderson DJ, Kaye KS, Chen LF, Schmader KE, Choi Y, Sloane R (2009). Clinical and financial outcomes due to methicillin resistant Staphylococcus aureus surgical site infection: a multi-center matched outcomes study. PLoS One.

[4] Bootsma MC, Diekmann O, Bonten MJ (2006). Controlling methicillin-resistant Staphylococcus aureus: quantifying the effects of interventions and rapid diagnostic testing. Proc Natl Acad Sci U S A.

[5] McBryde ES, Pettitt AN, McElwain DL (2007). A stochastic mathematical model of methicillin resistant Staphylococcus aureus transmission in an intensive care unit: predicting the impact of interventions. J Theor Biol.

[6] Chamchod F, Ruan S (2012). Modeling methicillin-resistant Staphylococcus aureus in hospitals: transmission dynamics, antibiotic usage and its history. Theor Biol Med Model.

[7] Mody L, Kauffman CA, Donabedian S, Zervos M, Bradley SF (2008). Epidemiology of Staphylococcus aureus colonization in nursing home residents. Clin Infect Dis.

[8] Garazi M, Edwards B, Caccavale D, Auerbach C, Wolf-Klein G (2009). Nursing homes as reservoirs of MRSA: myth or reality?. J Am Med Dir Assoc.

[9] Reynolds C, Quan V, Kim D, Peterson E, Dunn J, Whealon M (2011). Methicillin-resistant Staphylococcus aureus (MRSA) carriage in 10 nursing homes in Orange County, California. Infect Control Hosp Epidemiol.

[10] Manzur A, Gudiol F (2009). Methicillin-resistant Staphylococcus aureus in long-term-care facilities. Clin Microbiol Infect.

[11] Lee BY, Bartsch SM, Wong KF, Singh A, Avery TR, Kim DS (2013). The importance of nursing homes in the spread of methicillin-resistant Staphylococcus aureus (MRSA) among hospitals. Med Care.

[12] Barnes SL, Harris AD, Golden BL, Wasil EA, Furuno JP (2011). Contribution of interfacility patient movement to overall methicillin-resistant Staphylococcus aureus prevalence levels. Infect Control Hosp Epidemiol.

[13] Lesosky M, McGeer A, Simor A, Green K, Low DE, Raboud J (2011). Effect of patterns of transferring patients among healthcare institutions on rates of nosocomial methicillin-resistant Staphylococcus aureus transmission: a Monte Carlo simulation. Infect Control Hosp Epidemiol.

[14] Macal CM, North MJ, Collier N, Dukic VM, Wegener DT, David MZ (2014). Modeling the transmission of community-associated methicillin-resistant Staphylococcus aureus: a dynamic agent-based simulation. J Transl Med.

[15] Chamchod F, Ruan S (2012). Modeling the spread of methicillin-resistant Staphylococcus aureus in nursing homes for elderly. PLoS One.

[16] Murphy CR, Hudson LO, Spratt BG, Quan V, Kim D, Peterson E (2013). Predicting high prevalence of community methicillin-resistant Staphylococcus aureus strains in nursing homes. Infect Control Hosp Epidemiol.

[17] Tattevin P, Diep BA, Jula M, Perdreau-Remington F (2009). Methicillin-resistant Staphylococcus aureus USA300 clone in long-term care facility. Emerg Infect Dis.

[18] Otto M (2010). Basis of virulence in community-associated methicillin-resistant Staphylococcus aureus. Annu Rev Microbiol.

[19] Desai R, Pannaraj PS, Agopian J, Sugar CA, Liu GY, Miller LG (2011). Survival and transmission of community-associated methicillin-resistant Staphylococcus aureus from fomites. Am J Infect Control.

[20] Crnich CJ (2013). Impact and Management of MRSA in the Long-Term Care Setting. Curr Transl Geriatr and Exp GErontol Rep.

[21] Stone ND, Lewis DR, Johnson TM, Hartney T, Chandler D, Byrd-Sellers J (2012). Methicillin-resistant Staphylococcus aureus (MRSA) nasal carriage in residents of Veterans Affairs long-term care facilities: role of antimicrobial exposure and MRSA acquisition. Infect Control Hosp Epidemiol.

[22] Batina NG, Crnich CJ, Anderson DF, Dopfer D (2016). Models to predict prevalence and transition dynamics of methicillin-resistant Staphylococcus aureus in community nursing homes. Am J Infect Control.

[23] Crnich CJ, Duster M, Hess T, Zimmerman DR, Drinka P (2012). Antibiotic resistance in non-major metropolitan skilled nursing facilities: prevalence and interfacility variation. Infect Control Hosp Epidemiol.

[24] Keeling MJ, Rohani P (2008). Modeling infectious diseases in humans and animals.

[25] R Core Team. R: A language and environment for statistical computing.: R Foundation for Statistical Computing, Vienna, Austria. URL http://www.R-project.org/. 2014.

[26] Lawler GF (2006). Introduction to stochastic processes.

[27] Jackson CH (2011). Multi-State Models for Panel Data: The msm Package for R. J Stat Softw.

[28] Mody L, Kauffman CA, McNeil SA, Galecki AT, Bradley SF (2003). Mupirocin-based decolonization of Staphylococcus aureus carriers in residents of 2 long-term care facilities: a randomized, double-blind, placebo-controlled trial. Clin Infect Dis.

[29] Lee BY, Singh A, Bartsch SM, Wong KF, Kim DS, Avery TR (2013). The potential regional impact of contact precaution use in nursing homes to control methicillin-resistant Staphylococcus aureus. Infect Control Hosp Epidemiol.

[30] Diller R, Sonntag AK, Mellmann A, Grevener K, Senninger N, Kipp F (2008). Evidence for cost reduction based on pre-admission MRSA screening in general surgery. Int J Hyg Environ Health.

[31] Hacek DM, Paule SM, Thomson RB, Robicsek A, Peterson LR (2009). Implementation of a universal admission surveillance and decolonization program for methicillin-resistant staphylococcus aureus (MRSA) reduces the number of MRSA and total number of S. aureus isolates reported by the clinical laboratory. J Clin Microbiol.

[32] Gillespie DT (1976). A General Method for Numerically Simulating the Stochastic Time Evolution of Coupled Chemical Reactions. J Comput Phys.

[33] Gillespie DT (1977). Exact Stochastic Simulation of Coupled Chemical Reactions. J Phys Chem.

[34] Diekmann O, Heesterbeek H, Britton T. Mathematical Tools for Understanding Infectious Disease Dynamics. Princeton: Princeton University Press; 2013.

[35] Wasserman L (2004). All of statistics : a concise course in statistical inference.

[36] Embil J, Ramotar K, Romance L, Alfa M, Conly J, Cronk S (1994). Methicillin-resistant Staphylococcus aureus in tertiary care institutions on the Canadian prairies 1990-1992. Infect Control Hosp Epidemiol.

[37] Huang H, Flynn NM, King JH, Monchaud C, Morita M, Cohen SH (2006). Comparisons of community-associated methicillin-resistant Staphylococcus aureus (MRSA) and hospital-associated MSRA infections in Sacramento, California. J Clin Microbiol.

[38] Merrer J, Santoli F, Appere de Vecchi C, Tran B, De Jonghe B, Outin H (2000). “Colonization pressure” and risk of acquisition of methicillin-resistant Staphylococcus aureus in a medical intensive care unit. Infect Control Hosp Epidemiol.

[39] Cooper BS, Stone SP, Kibbler CC, Cookson BD, Roberts JA, Medley GF (2004). Isolation measures in the hospital management of methicillin resistant Staphylococcus aureus (MRSA): systematic review of the literature. BMJ.

[40] Bowler WA, Bresnahan J, Bradfish A, Fernandez C (2010). An integrated approach to methicillin-resistant Staphylococcus aureus control in a rural, regional-referral healthcare setting. Infect Control Hosp Epidemiol.

[41] Kauffman CA, Terpenning MS, He X, Zarins LT, Ramsey MA, Jorgensen KA (1993). Attempts to eradicate methicillin-resistant Staphylococcus aureus from a long-term-care facility with the use of mupirocin ointment. Am J Med.

[42] Currie A, Davis L, Odrobina E, Waldman S, White D, Tomassi J (2008). Sensitivities of nasal and rectal swabs for detection of methicillin-resistant Staphylococcus aureus colonization in an active surveillance program. J Clin Microbiol.

[43] Luteijn JM, Hubben GA, Pechlivanoglou P, Bonten MJ, Postma MJ (2011). Diagnostic accuracy of culture-based and PCR-based detection tests for methicillin-resistant Staphylococcus aureus: a meta-analysis. Clin Microbiol Infect.

[44] Temime L, Opatowski L, Pannet Y, Brun-Buisson C, Boelle PY, Guillemot D (2009). Peripatetic health-care workers as potential superspreaders. Proc Natl Acad Sci U S A.

[45] Obadia T, Silhol R, Opatowski L, Temime L, Legrand J, Thiebaut AC (2015). Detailed Contact Data and the Dissemination of Staphylococcus aureus in Hospitals. PLoS Comput Biol.

